# Improvement of Body Satisfaction in Older People: An Experimental Study

**DOI:** 10.3389/fpsyg.2019.02823

**Published:** 2019-12-12

**Authors:** Roberto Sánchez-Cabrero, Ana C. León-Mejía, Amaya Arigita-García, Carmen Maganto-Mateo

**Affiliations:** ^1^Department of Social Sciences and Applied Languages, Alfonso X el Sabio University, Madrid, Spain; ^2^Department of Psychology of Education and Psychobiology, International University of La Rioja (UNIR), Madrid, Spain; ^3^Department of Psychology, University of the Basque Country, San Sebastián, Spain

**Keywords:** body image, body satisfaction, older people, gender differences, body perception

## Abstract

Aging typically manifests itself in a variety of physical and cognitive alterations and challenges that are not always easily accepted. Feeling dissatisfied with these changes can also affect the mood and self-esteem of older people causing body image problems. The present study focuses on body satisfaction in Spanish older people (176 participants; *M* and *SD* = 64.03 ± 1 8.06; age range 50 to over 75) by employing experimental research to test whether psychosocial interventions may have a positive impact. Our aims are threefold: (1) To describe the body satisfaction of older people considering intervening variables, such as age, gender, having a stable partner, time of the year, and place of residence; (2) to compare body satisfaction improvement in older people participating in a specific body satisfaction program designed for this purpose versus a non-specific program run by the Spanish Red Cross; and (3) to examine the relationship between age, gender, having a stable partner, time of the year, place of residence, body satisfaction and participating in the experimental condition. The *IMAGINA* specific body image program yielded a significant improvement in body satisfaction when compared with the non-specific program in both men and women regardless of marital status and in some age groups: 50 to 54 years old, 60 to 64 years old, and 65 to 69. Male participants, as well as singles, were more satisfied with their bodies, and the contrary was true for divorced and separated. The *IMAGINA* program was particularly useful in participants with more body image problems. As shown, the pressure to fit beauty standards and related problems do not go away with age, a fact that is embodied and experienced differently in men and women.

## Introduction

The way we see our bodies influences how we feel about ourselves, and when our perceptions are negative, these can cause low self-esteem and mood problems. Body image could be defined as how a person sees, imagines, feels, and acts with his/her own body (Rosen, 1992; Thompson, 2004; Cash, 2017). We can distinguish two main elements: (1) a perceptive dimension that judges the size and proportions of one’s own body, and (2) a cognitive-emotional dimension that is commonly known as body satisfaction (Raich, 2004; Sánchez-Cabrero and Maganto, 2009; Maganto et al., 2016).

In this paper, we focus on body satisfaction, i.e., the subjective image of one’s own body in older people. This evaluation is regarded as unfavorable when it reduces personal confidence and makes someone feel bad about his/herself, and positive when it makes people feel good about themselves as well as comfortable when interacting with others (Tylka and Wood-Barcalow, 2015; Sánchez-Cabrero et al., 2019). Individuals in their fifties tend to experience body satisfaction problems as a result of bodily changes related to growing older (Hofmeier et al., 2017; Cameron et al., 2019). They particularly worry about aging signs such as wrinkles, hair loss, weakening of physical conditions, body odor, among others (Gubrium and Holstein, 2006; Longo, 2015; Vega et al., 2015). However, they are less concerned about weight, body shape, body composition (mass and fat proportions), and other specific worries typical in adolescents and young adults (Fernández-Bustos et al., 2015; Vega et al., 2015; Sabik and Versey, 2016; Irvine et al., 2019). As Clarke and Korotchenko (2011) pointed out, all aging signs are embodied, and it is not only in our mind that we experience age, but it is also through our bodies that we feel the psychosocial and physical consequences of growing older.

Across the lifespan, body image plays a vital role in self-concept and self-esteem. Body image problems have been associated in adolescence and youth with eating disorders, anxiety, and depression, but the psychological consequences of body dissatisfaction in older people are less known, and consequently there is a generational gap in research that needs to be filled (Deeks and McCabe, 2001; Tiggemann, 2004; Kilpela et al., 2015; Bouzas et al., 2019; Sánchez-Cabrero et al., 2019). The few studies that have addressed body image in both adulthood and old age have focused on female problems, particularly in menopausal-related issues (Deeks and McCabe, 2001; Webster and Tiggemann, 2003; Kilpela et al., 2015). For this reason, this study aims at filling this gap and providing data on body image dissatisfaction in maturity and old age.

Older people are inclined to accept their bodies better than teenagers do, and they are also less prone to developing eating and image disorders, yet this does not mean that they do not experience image problems at all (Webster and Tiggemann, 2003; McGuinness and Taylor, 2016). Indeed, the so-called *“maturity crisis”* is related, among other things, to how time takes its toll on physical appearance, making people feel sadder and disappointed in how they look (Gubrium and Holstein, 2006; Zdenko and Geiger-Zeman, 2015). Without a doubt, physical appearance decline is one of the most worrying signs that can make someone think of losing active lifestyles and see the possibility of developing physical and neurodegenerative diseases closer, and it can also awake thoughts of passing away. Not surprisingly, most people fear aging, and this becomes more patent at fifty, which makes this a significant age point. Despite being a psychosocial problem, particularly true of societies obsessed with youth, this has received little attention in the scientific literature (Tiggemann, 2004; Grogan, 2016; Cash, 2017). For instance, a systematic search in *PsycNet*, conducted in 2019 for this article, revealed that publications on body image focused on older people is less than the 5% of all articles published on this subject during the last 5 years.

Growing older does not only affect our perceptions of how we look, but it also affects the roles and social position that we identify ourselves with. In most modern societies, the status of older people is perceived to be lower and under-resourced. A significant number of retired people are very dependent on state support and retirement pensions to guarantee their primary living conditions. Therefore, the process of self-identification that takes place when someone feels that s/he is getting old implies a growing acceptance of new attributes and limitations. Sometimes these changes and adaptations come along with depressive symptoms or anxiety (Keyes and Westerhof, 2012). An intervention focused on body image could alleviate some of these problems by helping older people to keep positive attitudes and foster self-acceptance (Kozar, 2005; Mangweth-Matzek et al., 2006; McLean et al., 2011; Hudson et al., 2016; Mellor et al., 2017). In doing so, psychologists and mental health practitioners must become aware of the unique role that body satisfaction plays in accepting the self.

In the particular case of Spain, research on maturity and aging is even more necessary because demographically, it is one of the most aged countries worldwide (INE, Spanish Statistical and Office, 2019). And this trend will continue in the coming years, becoming a Spanish relevant social problem that needs psychosocial analysis and carefully planned interventions (Abellán et al., 2017).

Being satisfied with who we are is not only determined by our physical appearance but also by other variables. Particularly, by gender, age, place of residence (Swami et al., 2018), time of year and by whether we have a significant person or partner by our side (Markey et al., 2001; Davison and McCabe, 2005; Rodgers et al., 2015; Ridgway and Clayton, 2016). Regarding gender, older women tend to have less body satisfaction than men of the same age (Murray and Lewis, 2014; Tylka and Homan, 2015; Hudson et al., 2016; Cundall and Guo, 2017; Homan and Tylka, 2018). When compared with younger people, older people tend to experience less body dissatisfaction (Bucchianeri et al., 2014; Murray and Lewis, 2014), but this is not so clear as it has received little scholarly attention. As for having or not having a partner, being with someone is believed to have a positive impact on body satisfaction. However, Sánchez-Cabrero et al. (2019) found that the influence of marital status to body satisfaction could be mediated by gender in a more complex way, since married women were more dissatisfied with their body image in comparison with single women- In contrast, men were more satisfied regardless of their marital status (married or single). As can be seen, body satisfaction in older people could be the result of an intricate interaction between all these variables, and there is no evidence on how psychosocial interventions may also intervene in this process.

Currently, non-governmental organizations and public health services promote a variety of programs for older people trying to foster active aging. When it comes to body satisfaction, most of these interventions may be of little help since the scope of these programs is too broad, making it difficult to specifically address the acceptance of the body changes that come with age. The positive effects of participating in these non-specific programs may be due to the wellbeing experienced when relating to similar people, i.e., people of the same age, characteristics, and problems. In any case, it is difficult to know to what extent non-specific programs are truly effective and whether or not more specific programs on body satisfaction may be better instruments that we should rely on Castellano-Fuentes (2014), Roses-Gómez (2014), and Mellor et al. (2017).

As for intervention programs aimed at improving body image, the scientific literature shows that these produce outstanding results, from the classics of Cash (1997) and PICTA, Maganto et al. (2002), to current ones, such as those of, Kilpela et al. (2016), McCabe et al. (2017), and Bailey et al. (2019). However, none of them are intended for mature people, focusing instead on adolescent or young people, and mainly on the female population, except for the *IMAGINA* program by Sánchez-Cabrero (2012).

In this study, we examine body satisfaction in older people from an experimental perspective. Our aims are: (1) to describe body satisfaction of participants over 50 years old considering their age, gender, having a stable partner, time of the year, place of residence as intervening variables, and (2) To analyze body satisfaction improvement in older and mature people participating in a specific program on body satisfaction versus the improvement obtained in a non-specific program, and (3) to examine the relationship between age, gender, marital status, time of year, place of residence and body satisfaction regarding the experimental condition.

Regarding our first goal, the central hypothesis is that body satisfaction grows higher with age but not to the extent of making concern about physical appearance disappear (Webster and Tiggemann, 2003; McGuinness and Taylor, 2016; Sánchez-Cabrero et al., 2019). Regarding the second goal, the hypothesis is that, as suggested in previous literature on other age groups (Cash, 1997; Maganto et al., 2002; McCabe et al., 2017), the specific intervention on body image done in the experimental condition is going to have a positive influence on the evaluation of body satisfaction in the participants. This intervention consists of collective multi-thematic activities of a social and affective nature. Regarding the third goal that takes on the role of intervening variables in the experimental condition (gender, age, having a stable partner, time of the year, and place of residence), we hypothesize that gender is going to play a significant role in those participants above 50 years old (Tiggemann, 2004; Kilpela et al., 2015).

## Materials and Methods

### Participants

In this study, 176 people over 50 years old participated with no payment offered, half of them in the experimental condition, and the other half in the control condition. The mean age and standard deviation were 64.03 ± 18.06. Gender and age distributions were as follows, 30 men (17%) and 146 women (83%), 83 under 65 years of age (active population in terms of work), and 93 over 65 years of age (retired according to the Spanish labor system). There were 117 participants in a stable relationship, 37 widows/widowers, 15 singles, and only seven of them were separated or divorced from a long-term partner. Regarding the place of residence and the time of year, 113 lived in urban places, and 63 in rural areas, 92 of were enrolled in the program during summer and 84 during winter.

All the attributive variables measured in the study were categorical, and age was recorded in six different groups to make it easier to compare and analyze it with the rest of the variables of the study. The distribution of participants into six groups allow us to study the trend in the age range studied, while taking the 65 years old as the tipping point that marks the retirement from the labor market.

The participants were all recruited *via* the Spanish *Red Cross* in the North-West of Spain, which is one of the most affected areas by aging population problems. All of them were Caucasian (this Spanish region is not racially diverse), half of them came from rural places (localities with less than 1000 inhabitants) and the other half from urban areas. Sampling was done by clusters, having a total of 10 groups of people over 50 years old that were willing to participate in social programs of the Spanish Red Cross. Half of these groups underwent the experimental treatment, and the other half served as control. The programs were implemented by the same monitors at two different times of the year: summer and winter. The assignment of participants to the groups was random, ensuring similar distributions of them to the control and experimental conditions, and the same with the time of program application and place of residence.

### Data Obtaining Instruments

*Body Shape Questionnaire (BSQ)* developed by Cooper et al. (1987), which was adapted and scaled to Spanish participants by Raich et al. (1996). This is a self-report of 34 items following a Likert scale that goes from 1 (never) to 6 (always). The final score ranges from 34 to 204, and scoring above 110 indicates dissatisfaction and discomfort with physical appearance (Cooper et al., 1987). It is a reliable instrument since several studies have reported Cronbach’s α between 0.95 and 0.97. In this study, Cronbach’s alpha was 0.96, so it is near the values obtain by Cooper et al. (1987). Also, the *BSQ* has good external validity, i.e., it is convergent with other similar tools, such as the *Multidimensional Body Self-Relations Questionnaire, MBSRQ* (Cash, 2015) and the body dissatisfaction subscale of the *Eating Disorders Inventory*, *EDI* (Garner et al., 1983).

We used the *BSQ* because it continues to be one of the most widely used instruments in scientific research (Baile et al., 2002; Fernández-Bustos et al., 2015) and it remains a benchmark in body image studies. Proof of this is recent and cultural adaptations, for instance, to the Brazilian population (Conti et al., 2009), to the Colombian (Moreno et al., 2015), to the Norwegian (Kapstad et al., 2015) or the Korean (Kim and Chee, 2018), among others. Also, it has excellent psychometric properties and has been already adapted to Spanish. Besides, the lexical simplicity and brevity of its application make it the right choice for older people. Let us note that due to their age, the participants could get tired within a few minutes of evaluation, and that, in some cases, had low literacy skills.

Although older people tend to worry about aging signs, weight, and body changes, using the BSQ allows us to compare our results on aging people with other studies on younger participants, making it possible to examine body dissatisfaction across the lifespan. An ultimate reason for using BSQ is that currently, we do not have body image questionnaire specific for older people, and creating a new instrument would cause reliability problems, precluding us from comparing our results with existing literature.

Information about sociodemographic variables such as age, gender, and marital status was also collected *via* questionnaire. In contrast, the details about the time of year of program application, and place of residence was registered by the person who gathered and controlled the experimental data.

The BMI (Body Mass Index) was not evaluated since possible misinterpretations can occur due to the gradual shrinkage of the spine that occurs in older people that makes the BMI seem higher than it is. More importantly, in Spain (and particularly in rural areas), asking about this information can be seen as an intrusion on personal issues. We excluded from the study people with chronic severe health problems, obesity-related serious conditions, or extreme thinness to avoid any bias in the results.

As for the treatment of the experimental group, we used the *IMAGINA* Program that was specifically designed to improve the body image of mature and older adults in the Spanish population (Sánchez-Cabrero, 2012). It consists of eight sessions of 90–120 min to do in groups. The entertaining activities aim at improving the body image and self-esteem of participants, encouraging social participation, appearance acceptance, and healthy nutrition. It also enhances emotional intelligence, it promotes positive affective relations with people, and it helps re-evaluate self-expectations related to physical appearance and social interaction that can be harmful to the mental health of the participant. This program is unique in its field, and therefore the best option to study body image improvement of the participants in this study. In the satisfaction survey that participants completed in the pilot test, the program had an excellent acceptance (was rated 9 out of 10).

As a control treatment, we used the *“Spanish Red Cross Health Promotion Program for the older people”* that had an excellent acceptance among its participants. It has the same duration as *IMAGINA* (eight sessions of 90–120 min), and it was also designed for groups. Whereas this standard program fostered social interaction, it did not address the body image issues that *IMAGINA* did. Participants enjoyed group leisure activities and were trained in healthy habits.

Both treatments were carried out in the evening, twice a week, during a total of 4 weeks. The *BSQ* was not applied to those participants who did not attend every single session. Consequently, data from 10% of the participants were not included in the study. Since both programs were applied in groups, enjoying playful activities and positive social participation, the experimental attrition or mortality of both groups was reduced to a minimum.

### Design and Procedure

We followed an experimental design to evaluate inter and intra-specific interactions between body image satisfaction (DV) and program participation: body image specific program vs. non-specific social program (intergroup or between subjects’ IV effect), and before and after participating (intragroup or within subjects’ IV effect). We controlled blocking variables through sample selection and randomization of the subjects into the experimental conditions. We also assessed the influence and interaction of age, gender, marital status, time of year, and place of residence as part of the study’s goals.

There was a total of ten groups (five participating in the specific body image program and five in the non-specific program). Participants were allocated randomly to the control and experimental conditions. The experimental group participated in the “IMAGINA: Program to improve the self-esteem and body image in adults” (Sánchez-Cabrero, 2012), taking part in eight group sessions of 2 h each (two times per week, a total of 4 weeks). The control group participated in a non-specific psychosocial program implemented by the Spanish Red Cross, also with eight sessions of 2 h duration each.

The BSQ was applied at two different times, before and after the experimental treatment as follows: (1) pretest with the groups already formed and before participating in their assigned program; (2) posttest immediately after the end of the last session of the program. The application of BSQ was in paper format and done individually, although all participants of each group were in the same room at the same time. Each measurement had a proximate duration of 10 min to respond to 34 items.

According to the *Helsinki Declaration* (World Medical Association, 2013), we followed ethical principles for psychological research strictly. We informed all participants of the purpose of the study and that it had a non-profit nature and was non-sponsored, and gathered their consent to take part in the research afterward. The older people are vulnerable and, therefore, we made sure that they all understood our purposes and the possible benefits of participating in both programs correctly. It was possible to withdraw from the study, but none of the participants withdrew. The Institutional Review Board at Alfonso X el Sabio University approved the experimental protocol.

### Data Analyses

To test our research hypotheses, we conducted descriptive and inferential analyses, more specifically, a paired samples *Student’s t-test* to examine body image before and after participating in the two programs (intragroup IV effect), and a *One-way ANOVA* to assess the effect of each program (intergroup IV effect), sex, age, and marital status. This analysis was completed with *repeated measures ANOVA* using the *Pillai’s Trace* and *Wilks’ Lambda* statistics, as they offer opposite and complementary results of the inter-intragroup effect of independent variables. We considered that assessing variances and covariances sphericity in the multivariate analyses was not necessary because there only were two intra levels, and thus, the covariance is equal to itself.

## Results

Table 1 shows descriptive analyses of the *BSQ* test in both conditions (experimental and control) before and after participants took place in them, and the difference between these two moments (*paired samples test)*.

**TABLE 1:** Means and standard deviations of the BSQ test in both conditions and moments (pre, post) and pre-post-test difference (paired samples test).

**Experimental group (*n* = 88)**								**Control group (*n* = 88)**							
**Pre-test**		**Post-test**		**Pre-post**				**Pre-test**		**Post-test**		**Pre-post**			
***M***	***SD***	***M***	***SD***	***M***	***SD***	***p***	***Cohen’s d***	***M***	***SD***	***M***	***SD***	***M***	***SD***	***p***	***Cohen’s d***
71.9	24.2	65.1	21.4	6.75	9.34	0.000	0.721	69.2	22.7	68.5	20.9	0.75	7.97	0.380	0.094

The results of the paired samples test (intragroup effect) indicate that the improvement is higher in the *IMAGINA* body image program than in the non-specific intervention (*M* = 6.75 *versus M* = 0.75), and this result is statistically significant in the experimental condition (*t* = 6.782, *p* = 0.000). Besides, the improvement is not significant in the control condition alone (*t* = 0.883, *p* = 0.380), which is an indication of a noteworthy improvement related to the *IMAGINA* body image program versus the non-specific intervention (*Cohen’s d* = *0.721 versus* 0.94). However, it is necessary to use multivariate analyses, such as the *Repeated Measures ANOVA*, to confirm whether the effect of *IMAGINA* was higher than the non-specific program or if, on the contrary, these differences were due to chance. In this sense, Table 2 shows a *One-Way ANOVA* (intergroup effect), which compares *BSQ* at both moment (pre and post-treatment) and both conditions (control vs. experimental) as well as the difference between these two moments (pre-post difference). Both pre (*F* = 0.56, *p* = 0.455) and post-condition (*F* = 1.108, *p* = 0.294) show non-significant mean differences between the experimental and control conditions. However, there is a significant improvement in *BSQ* in the pre-post difference (*F* = 21.019, *p* = 000).

**TABLE 2:** BSQ differences in both moments (One-way ANOVA between experimental conditions).

**Pre-test**			**Post-test**			**Pre-post**		
***F***	***p***	**Eta squared**	***F***	***p***	**Eta squared**	***F***	***p***	**Eta squared**
0.56	0.455	0.003	1.108	0.294	0.006	21.019	0.000	0.107

Finally, in Table 3, we can see the results of the *repeated measurements ANOVA* (inter-intra group effect), that also points to the effectiveness of the *IMAGINA* body satisfaction program over the non-specific one since all the multivariate contrasts are statistically significant (*p* = 0.000).

**TABLE 3:** Multivariate test.

**Effect**	**Statistical tools**	**Value**	***F***	**Gl. hyp.**	**Gl error**	***p***	**Partial Eta Squared**
BSQ differences between the PRE and POST test	Pillai’s Trace	0.16	32.84	1	174	0.000	0.159
	Wilks’ Lambda	0.84	32.84	1	174	0.000	0.159
Impact of the variable “Condition” (inter) over the PRE and POST treatment measurement of the BSQ test (intra)	Pillai’s Trace	0.11	21.02	1	174	0.000	0.108
	Wilks’ Lambda	0.89	21.02	1	174	0.000	0.108
Intercept	*MS* = 1659627.56		1729.82	1	174	0.000	0.909

*MS, mean square.*

### Gender, Age, Marital Status, Time of Year and Place of Residence Differences

Table 4 shows the mean and standard deviations of *BSQ* in both conditions and moments (pre and post) by gender, age marital status, time of year, and place of residence, as well as the difference between these two moments (*paired samples test).*

**TABLE 4:** Age, gender, marital status, time of year, and place of residence differences (paired samples test).

	**Experimental group (*n* = 88)**				**Control group (*n* = 88)**			
	**Pre-test**	**Post-test**	**Pre-post**		**Pre-test**	**Post-test**	**Pre-post**	
	***M (SD)***	***M (SD)***	***M (SD)***	***p (Cohen’s d)***	***M (SD)***	***M (SD)***	***M (SD)***	***p (Cohen’s d)***
**Gender**								
WOMAN (*n* = 146)	71.9 (25.2)	65.3 (22.0)	6.6 (9.85)	(0.000) 0.673	72.3 (21.4)	71.3 (19.8)	1.07 (8.29)	(0.277) 0.129
MAN (*n* = 30)	71.5 (18.8)	63.9 (18.2)	7.57 (6.1)	(0.000) 1.239	55.2 (24.0)	55.9 (21.6)	−0.69(6.37)	(0.672) 0.109
**Age**								
From 50 to 54 (*n* = 30)	78.3 (28.6)	66.6 (23.3)	11.74 (10.89)	(0.000) 1.076	80.1 (25.9)	78.0 (23.0)	2.09 (6.11)	(0.283) 0.346
From 55 to 59 (*n* = 22)	87.9 (25.1)	80.1 (2.9)	7.71 (10.13)	(0.090) 0.763	72.9 (20.9)	73.3 (23.2)	−0.33(6.33)	(0.841) 0.054
From 60 to 64 (*n* = 31)	72.8 (23.1)	68.5 (19.5)	4.31 (7.52)	(0.037) 0.575	75.0 (25.5)	76.3 (20.7)	−1.33(8.72)	(0.563) 0.152
From 65 to 69 (*n* = 47)	72.1 (24.8)	65.5 (24.5)	6.55 (7.92)	(0.001) 0.822	72.0 (20.5)	69.2 (17.9)	2.72 (9.51)	(0.166) 0.286
From 70 to 74 (*n* = 27)	64.9 (15.8)	59.3 (15.1)	5.64 (10.29)	(0.061) 1.034	57.6 (18.6)	58.4 (15.4)	−0.77(8.16)	(0.740) 0.089
Over 75 (*n* = 19)	56.1 (16.6)	53.6 (16.7)	2.5 (7.62)	(0.327) 0.328	49.2 (12.4)	48.1 (12.1)	1.11 (6.41)	(0.617) 0.173
**Marital status**								
In couple (*n* = 117)	73.8 (23.4)	67.3 (19.9)	6.53 (10.12)	(0.000) 0.647	71.3 (23.7)	69.9 (21.8)	1.35 (8.23)	(0.209) 0.164
Separated (*n* = 7)	104.7 (25.4)	92.0 (25.4)	12.67 (4.51)	(0.040) 2.787	89.7 (15.9)	88.2 (15.6)	1.5 (4.43)	(0.547) 0.339
Single (*n* = 15)	63.2 (15.8)	56.2 (16.7)	7 (6.51)	(0.008) 1.075	49.0 (10.0)	52.8 (12.3)	−3.8(5.36)	(0.188) 0.708
Widow/widower (*n* = 37)	65.1 (26.2)	58.8 (24.1)	6.75 (9.34)	(0.007) 0.719	63.6 (18.3)	63.7 (16.8)	−0.11(8.24)	(0.956) 0.012
**Time of year**								
Summer (*n* = 92)	72.1 (21.2)	67.7 (20.0)	4.40 (9.46)	(0.003) 0.465	70.2 (22.5)	69.4 (20.1)	0.78 (8.93)	(0.562) 0.088
Winter (*n* = 84)	71.6 (27.5)	62.2 (22.8)	9.44 (8.54)	(0.000) 1.104	68.2 (23.1)	67.5 (21.8)	0.72 (6.93)	(0.499) 0.104
**Place of residence**								
Rural (*n* = 63)	70.2 (18.4)	66.0 (19.1)	4.21 (8.69)	(0.008) 0.484	65.6 (20.6)	64.6 (17.8)	0.93 (9.28)	(0.593) 0.100
Urban (*n* = 113)	72.9 (27.3)	64.6 (22.9)	8.35 (9.45)	(0.000) 0.887	71.0 (23.6)	70.3 (22.1)	0.66 (7.33)	(0.491) 0.090

Regarding gender, male participants were more satisfied with their bodies than females, and this difference is even more evident after taking part in the *IMAGINA* program (post-test). Moreover, the pre-post difference is statistically significant for both men and women only in the experimental condition (Women: *t* = 5.756, *p* = 0.000; Men: *t* = 4.646, *p* = 0.000).

As for the participant’s age, since they have been classified into 6 groups, we applied Bonferroni’s corrections to avoid the risk of error type I when making multiple comparisons. This way, the result is only considered significant when *p* is less than 0.08. Body satisfaction improved in all groups for the experimental condition, but this result was only significant for participants 50 to 54 years old (*t* = 4,70; *p* = 0.000) and 65 to 69 (*t* = 1.038; *p* = 0.001). Again, there were no differences in any of the groups of the control condition.

Regarding marital status (recorded into four groups), we applied Bonferroni’s corrections to avoid the risk of error type I when making multiple comparisons, considering a result significant when *p* is below 0.125. These results are affected by the small sample size of some of the groups, such as separated or divorced (*n* = 7) and singles (*n* = 15). Separated and divorced individuals are less satisfied with their image before participating, and this was true in both conditions (*M* = 104.07; *SD* = 25.4 in the experimental group; *M* = 89.7; *SD* = 15.9 in the control group), which seems to indicate that their marital status is negatively related to their body satisfaction. Despite this unfavorable initial result, it is the group that improves more when participating in the *IMAGINA* program (*M* = 12.7; *Cohen’s d* = 2.787), yet these results are not significant (*p* = 0.04). As for the singles, they are the ones with the highest initial body satisfaction levels (*M* = 63.2; *SD* = 15.8 in experimental group; *M* = 49; *SD* = 10 in the control group). However, taking part in the control program seems to be a negative experience for them since their body satisfaction scores measured in the pre-post difference are negative (−3.8), although this is not a significant result (*p* = 0.188). The only groups whose sample size was acceptable was that of participants with stable relationships, i.e., in a couple (*n* = 117) and widows/widowers (*n* = 37). In these cases, the *IMAGINA* body program had a positive impact as they improved their satisfaction significantly (*p* = 0.000 and *p* = 0.007, respectively).

The time of year at which the program took place did not affect the control group significantly, but it did the experimental group. In the experimental group, there were higher scores in winter than in summer (*M* = 9.44 and *M* = 4.40, respectively), although the improvement was significant in both seasons *p* = 0.003 and *p* = 0.000, respectively.

Finally, regarding the place of residence, there were differences between both groups since the improvement was higher in urban participants (*M* = 8.35) than in rural participants (*M* = 4.21) in the experimental condition. The size of the effect is only significant in the experimental condition, as it happens with the rest of the attributive variables. Again, there were no significant differences in any of the groups of the control condition.

As can be seen, age, gender, time of year, and place of residence have less effect on body satisfaction in control groups than in the *IMAGINA* program groups, as shown by the *Cohen’s d*. More specifically, if we look at the effect of the intergroup (IV) in Table 5, we can see how the results obtained in the *One-Way ANOVA* confirm that most of the significant differences are in the pre-post differences. Besides, the effect size of pre-post difference (*Eta Squared*) is bigger than the pre and post measures alone.

**TABLE 5:** BSQ inter analysis by age, gender, marital status, time of year and place of residence across conditions (One-way ANOVA).

	**Pre-test**			**Post-test**			**Pre-post**		
	***F***	***p***	**Eta squared**	***F***	***p***	**Eta squared**	***F***	***p***	**Eta squared**
**Gender**									
WOMAN	0.011	0.918	0.000	2.918	0.09	0.019	13.4	0.000	0.085
MAN	4.203	0.050	0.131	1.2	0.283	0.041	13.07	0.001	0.318
**Age**									
From 50 to 54	0.029	0.667	0.001	1.683	0.205	0.056	7.24	0.012	0.205
From 55 to 59	2.154	0.158	0.097	0.444	0.513	0.021	5.253	0.033	0.208
From 60 to 64	0.063	0.804	0.002	1.176	0.287	0.038	3.741	0.063	0.114
From 65 to 69	0.000	0.984	0.000	0.355	0.554	0.007	2.209	0.144	0.046
From 70 to 74	1.223	0.279	0.046	0.024	0.879	0.000	3.188	0.086	0.113
Over 75	1.025	0.326	0.056	0.659	0.428	0.037	0.182	0.675	0.010
**Marital status**									
In couple	0.326	0.569	0.002	0.482	0.489	0.004	9.263	0.003	0.074
Separated	0.928	0.38	0.156	0.06	0.816	0.012	5.253	0.022	0.682
Single	3.285	0.093	0.202	0.162	0.694	0.012	10.17	0.007	0.439
Widow/widower	0.04	0.843	0.001	0.532	0.47	0.015	5.281	0.028	0.131
**Time of year**									
Summer	0.167	0.684	0.001	0.178	0.674	0.002	3.568	0.062	0.038
Winter	0.388	0.535	0.004	1.173	0.282	0.014	26.529	0.000	0.244
**Place of residence**									
Rural	0.882	0.351	0.014	0.083	0.775	0.001	2.088	0.154	0.033
Urban	0.158	0.692	0.001	1.867	0.175	0.017	23.583	0.000	0.175

Finally, Table 6 shows the multivariate analyses with repeated measures (inter-intragroup effect) that indicate that age, gender, marital status, time of year, and place of residence do not interfere with the effectiveness of the treatment (*IMAGINA* program) as the effect is non-significant.

**TABLE 6:** Multivariate contrasts of gender, age, gender, marital status, time of year, and place of residence (inter and intra analyses).

**Effect**	**Statistical tool**	**Value**	***F***	**Gl. hip.**	**Gl. error**	***p***	**Partial Eta Squared**
Impact of the variable Gender (inter) over the PRE and POST treatment measurement of the BSQ (intra) test	Pillai’s Trace	0.00	0.05	1	172	0.824	0.000
	Wilks’ Lambda	1.00	0.05	1	172	0.824	0.000
Impact of the variable Gender (inter) over the PRE and POST treatment measurement of the BSQ (intra) test keeping in mind the variable Condition (inter)	Pillai’s Trace	0.00	0.61	1	172	0.436	0.004
	Wilks’ Lambda	1.00	0.61	1	172	0.436	0.004
Intercept BSQ-Gender	*MS* = 861914.63		926.61	1	172	0.000	0.843
Impact of the variable Age (inter) over the PRE and POST treatment measurement of the BSQ (intra) test	Pillai’s Trace	0.05	1.60	5	164	0.163	0.047
	Wilks’ Lambda	0.95	1.60	5	164	0.163	0.047
Impact of the variable Age (inter) over the PRE and POST treatment measurement of the BSQ (intra) test keeping in mind the variable Condition (inter)	Pillai’s Trace	0.02	0.72	5	164	0.606	0.022
	Wilks’ Lambda	0.98	0.72	5	164	0.606	0.022
Intercept BSQ-Age	*MS* = 1443252.91		1652.99	1	172	0.000	0.910
Impact of the variable Marital status (inter) over the PRE and POST treatment measurement of the BSQ (intra) test	Pillai’s Trace	0.01	0.68	3	168	0.564	0.012
	Wilks’ Lambda	0.99	0.68	3	168	0.564	0.012
Impact of the variable Marital status (inter) over the PRE and POST treatment measurement of the BSQ (intra) test keeping in mind the variable Condition (inter)	Pillai’s Trace	0.01	0.62	3	168	0.599	0.011
	Wilks’ Lambda	0.99	0.62	3	168	0.599	0.011
Intercept BSQ-Marital Status	*MS* = 621802.63		695.612	1	172	0.000	0.805
Impact of the variable Time of year (inter) over the PRE and POST treatment measurement of the BSQ (intra) test	Pillai’s Trace	0.02	3.72	1	172	0.055	0.021
	Wilks’ Lambda	0.98	2.04	1	172	0.055	0.021
Impact of the variable Time of year (inter) over the PRE and POST treatment measurement of the BSQ (intra) test keeping in mind the variable Condition (inter)	Pillai’s Trace	0.02	3.90	1	172	0.051	0.022
	Wilks’ Lambda	0.98	3.90	1	172	0.051	0.022
Intercept BSQ-Time of Year	*MS* = 1652369.59		1708.12	1	172	0.000	0.909
Impact of the variable Place of residence (inter) over the PRE and POST treatment measurement of the BSQ (intra) test	Pillai’s Trace	0.01	2.04	1	172	0.155	0.012
	Wilks’ Lambda	0.99	2.04	1	172	0.155	0.012
Impact of the variable Place of residence (inter) over the PRE and POST treatment measurement of the BSQ (intra) test keeping in mind the variable Condition (inter)	Pillai’s Trace	0.02	2.65	1	172	0.106	0.015
	Wilks’ Lambda	0.98	2.65	1	172	0.106	0.015
Intercept BSQ-place of residence	*MS* = 1496025,23		1552,66	1	172	0.000	0.900

*MS, mean square.*

## Discussion

Our results show that those participants who partook in the *IMAGINA* body image program improved their satisfaction toward their body look, whereas those who participated in a non-specific program did not experience any significant improvement. The score differences between the two conditions (6.75 points of improvement in the experimental group vs. 0.75 points in the control condition, *p* = 0.000) are in line with what positive body image theory claims, i.e., that self-image satisfaction can be fostered (Tylka and Homan, 2015; Homan and Tylka, 2018). This experimental study is also coherent with other studies suggesting that tailored programs with specific scopes are better instruments than preventive interventions that are non-specifically orientated (Roses-Gómez, 2014; Kilpela et al., 2015; Hudson et al., 2016; McCabe et al., 2017; Mellor et al., 2017; Bailey et al., 2019). Besides, our results also show the goodness of design of the *IMAGINA* program by Sánchez-Cabrero (2012) when used with participants over 50 years old.

The above results have clear clinical and scientific implications, as they show that we can intervene on body image at all ages and that physical appearance continues to be a relevant concern in the last phases of life. Moreover, the inclusion of body image intervention on mature and aging people can be a complementary action to the medical attention given to patients who are suffering depressive or altered moods. In this sense, it would be desirable to explore further the benefits of body satisfaction intervention on mature or older people and its possible benefits on mental health in both a preventive and palliative way. Also, we should look scientifically at the process of body image satisfaction in old age, determining its conditioning, limiting, and positive effects on mood and social interaction.

In our society, image and physical appearance are matters of great social importance due to the role that media usage (e.g., Internet, TV, magazines) plays in appearance comparisons, meaning that we are constantly exposed to “ideals” of beauty, health and fitness to live up to Fardouly et al. (2015) and Ridgway and Clayton (2016). This also happens in those groups who are not directly targeted by these media portrayals, such as older people (Raich, 2004; Thompson, 2004; Cash, 2017; Mellor et al., 2017). Despite its social and health relevance, body satisfaction has not received much scholarly attention, particularly in Spain, where there are no similar studies. For this reason, it is hard to establish any comparison. However, our participants’ scores on body satisfaction assessed with the *BSQ* are similar to those reported in previous studies validating this tool. More specifically, we found a score of 71.9 and 72.3 (experimental and control group, respectively) for women, which is lower but in line with the 84.7 of Cooper et al. (1987) and the 84.75 of Raich et al. (1996). In the 21st century, the study of Baile et al. (2002) with more than five hundred preadolescents found a score of 81.2 among girls between 15 and 16 years old, and 79.49 for girls between 17 and 19. Another recent study by Conti et al. (2009), showed an average of 73.9 among Brazilian adolescents, 88.3 for women and 57.1 for men, reflecting a very pronounced gender difference. Finally, the latest study of Fernández-Bustos et al. (2015) conducted with more than five hundred female teenagers and pre-teenagers also reported similar values, yet no specific mean and standard deviation values were provided. If we compare these results with the ones obtained in this study with people over 50 years old (*M* = 70.54; *SD* = 23.44). We can see that even though there are differences between age-groups, the range that includes the 68% of the data (i.e., the area around one SD over the mean) overlaps in more than 60% of the cases. Thus, we could conclude that most of the people over 50 years old may have similar body dissatisfaction levels as teenagers and young adults.

Our results also support the conclusion of other studies showing how concerns about physical appearance do not disappear as people age (McGuinness and Taylor, 2016; Bouzas et al., 2019; Cameron et al., 2019). More specifically, we refer to the study of Mangweth-Matzek et al. (2006) and the longitudinal study of McLean et al. (2011). As Gubrium and Holstein (2006) hypothesized, older people may perceive their appearance as a *“Mask”* of their *“True”* physical self that was captured by the pictures taken in their youth. Such disconnection or non-identification with the current physical self could alter their perception, making them more dissatisfied with their body image. Consequently, body image does play a significant role in emotional health (i.e., self-esteem and self-concept) and life satisfaction (Castellano-Fuentes, 2014; Vega et al., 2015; Zdenko and Geiger-Zeman, 2015). Therefore, more specific body image programs like *IMAGINA* (Sánchez-Cabrero, 2012), presented here, should be developed and promoted by governments and non-governmental organizations (Tiggemann, 2004; Kilpela et al., 2015; Bouzas et al., 2019).

Even though age is positively related to body satisfaction, it has been suggested that it only matters if other social and biological factors are present, such as losing social expectations or perceiving that physical decline is occurring (Deeks and McCabe, 2001; Kozar, 2005; Hofmeier et al., 2017). Our data showed that although body satisfaction tends to improve with age, it goes down between 55 and 59 years, a period in which there is a peak in body dissatisfaction that cannot be explained by other attributive variables. However, these differences are not significant for any age group and they may be due to chance. On the other hand, the improvement in body satisfaction that happens in the experimental group is significantly higher in the period of 65 to 69 years. This effect is probably due to the retirement process, which quantitatively and qualitatively transforms the social interactions of older people, making the positive effect of participating in the body image program more salient. In light of these results, we need new and more specific studies about the role of age in body satisfaction of older people.

Interestingly, in our study, we found that growing older do not eliminate the concern for physical appearance, which happens to be strongly related to gender. A fact that is line with what other scholars have suggested when highlighting that the body is sexed and that it is such sexualized embodiment that determines which features and attributes are perceived as belonging to the self-image (Longo, 2015; Cash, 2017; Cundall and Guo, 2017). For instance, according to traditional standards, female and male traits arise from an active-passive polarity (strength, endurance, energy, audacity for males, and their opposites for females) that influences how we judge our body (Raich, 2004; Sánchez-Cabrero and Maganto, 2009; Sánchez et al., 2015). Also, our results showed that men were more satisfied with their body image, and this was true for both conditions. Gender differences could be explained in terms of social exposure and judgments, which make men realize that as they age, they lose the *“active”* traits mentioned above, becoming less masculine (Rodgers et al., 2015; Cash, 2017). This is compatible with the fact that women are more dissatisfied with their body image and critical with themselves across the lifespan (Mellor et al., 2010; Grogan, 2016). Also, according to Davison and McCabe (2005), body satisfaction in men is more linked to affective and sexual factors (inter competition), whereas in women, it is more related to social qualities and interaction with other women (intra competition). However, these gender results should be addressed with caution, since the difference in the number of female (*n* = 146) and male participants (*n* = 30) is considerable and may have biased the results. This undesired situation was due to the higher interest of women in participating in social group programs, a gender difference that reflects social reality. Besides, the risk of experimental attrition for 8 weeks is high, and therefore, we could not afford to wait for having more men to enroll with us.

In general, our results show that singlehood is positively related to body image (*M* = 58.47 in *BSQ*; Women = 63.2 and Men = 52.8) and that people more dissatisfied were separated or divorced (*M* = 96.14 in *BSQ*; Women = 104.7; Men = 89.7) as well as people with partners (*M* = 72.51 in *BSQ*; Women = 73.8; Men = 71.3). It is worth noting that the *IMAGINA* program benefited the participants who, according to their satisfaction level, needed the most help (separated and divorced people).

Finally, the last two variables evaluated, time of the year and place of residence, behave similarly. There are no clear differences in body satisfaction, but there are in how *IMAGINA* program affects: being urban or participating in *IMAGINA* during winter doubles its positive effects. About the season, the body silhouette is more hidden under the voluminous warming clothing that people use in winter, making them feel better or less concerned about how they look. As for the place of residence, in urban areas there may be fewer social interaction opportunities for older people, and hence, the effects of the social activities of the program may be more salient.

This study is not exempt from limitations, the main one being that there are no similar studies to compare it with and, therefore, more replications are needed to validate our results. Also, we only measured body satisfaction and did not include other related variables, but this was because the program whose efficacy we wanted to analyze had a strong focus on this variable.

Another relevant limitation is the lack of body image programs conducted in maturity and old age, which has limited the treatment only to a single alternative. However, *IMAGINA* has proved to be very useful in its work, as shown by the results obtained.

Finally, two notable shortcomings regarding the selection of variables are worth mentioning. Firstly, due to the place in which the study took place, all participants were white, and ethnicity could not be analyzed. Secondly, we decided not to use the BMI because when aging, the spine curve reduces the height of the older person, and therefore, the BMI may not be a reliable indicator. Also, asking for this information is culturally problematic and can be seen as invasive, generating discomfort in the participants.

To conclude, mature and older people are concerned with their image and suffer from not fitting in with the beauty and health standards that our current society impose on all of us. This is true for men and women, yet it is experienced differently and depends as well on social variables like marital status. Let us not forget that older people struggle to accept their individuality in a society that celebrates youth and dislike aging, being different, or not fitting the beauty standards. This concerns the media and advertising sector in which more psychosocial research and actions are needed since too often they are reaching at-risk groups (i.e., children and older people) that were not meant to be influenced by their advertising and marketing content.

## Data Availability Statement

The datasets generated for this study are available on request to the corresponding author.

## Ethics Statement

The study was conducted according to the Declaration of Helsinki and approved by the Institutional Review Board at Alfonso X el Sabio University. Written informed consent was obtained from all participants of this study.

## Author Contributions

RS-C led the research, analyzed the data, and wrote the main body of the manuscript. AL-M reviewed the data, translation, and experimental design presented in the reports of this manuscript. AA-G and CM-M reviewed and corrected the statistical analyses of the results, and reviewed the bibliography. All authors reviewed the final manuscript.

## Conflict of Interest

The authors declare that the research was conducted in the absence of any commercial or financial relationships that could be construed as a potential conflict of interest.
